# A Stack-based Ensemble Framework for Detecting Cancer MicroRNA Biomarkers

**DOI:** 10.1016/j.gpb.2016.10.006

**Published:** 2017-12-12

**Authors:** Sriparna Saha, Sayantan Mitra, Ravi Kant Yadav

**Affiliations:** Department of Computer Science and Engineering, Indian Institute of Technology, Patna 801103, India

**Keywords:** Sequential minimal optimizer, Non-dominated sorting genetic algorithm, Multiobjective optimization, MicroRNA

## Abstract

**MicroRNA** (miRNA) plays vital roles in biological processes like RNA splicing and regulation of gene expression. Studies have revealed that there might be possible links between oncogenesis and expression profiles of some miRNAs, due to their differential expression between normal and tumor tissues. However, the automatic classification of miRNAs into different categories by considering the similarity of their expression values has rarely been addressed. This article proposes a solution framework for solving some real-life classification problems related to cancer, miRNA, and mRNA expression datasets. In the first stage, a **multiobjective optimization** based framework, **non-dominated sorting genetic algorithm** II, is proposed to automatically determine the appropriate classifier type, along with its suitable parameter and feature combinations, pertinent for classifying a given dataset. In the second page, a stack-based ensemble technique is employed to get a single combinatorial solution from the set of solutions obtained in the first stage. The performance of the proposed two-stage approach is evaluated on several cancer and RNA expression profile datasets. Compared to several state-of-the-art approaches for classifying different datasets, our method shows supremacy in the accuracy of classification.

## Introduction

Cancer is one of the leading causes of death in the world [1], [2], making it imperative to detect cancer in the early stages for proper diagnosis. MicroRNAs (miRNAs) have been reportedly linked with various types of cancers and expressed differentially in tumor versus normal tissues [3], [4], [5]. However, most of miRNA studies [6], [7], [8], [9], [10], [11], [12], [13] focus on biological aspects. miRNAs can be categorized into normal and tumor types depending on their expression levels. Therefore, automatic classification of a miRNA sample into any of these two classes is a pressing problem.

Many supervised machine learning algorithms have been developed for data classification, which take labeled data as input and produce inference for mapping the unknown data [14], [15]. These algorithms comprise of several parameters whose values can be changed according to the problem at hand, thus improving the classification performance. For instance, Gaspar-Cunha et al. [16] developed an algorithm named Reduced Pareto Set Genetic Algorithm with elitism (RPSGAe) by combining a multiobjective based optimization framework [17] with support vector machine (SVM) [18] for automatic classification of single proton emission computed tomography (SPECT) data. Peng et al. [19] used SVM-based recursive feature elimination technique (SVM-nRFE) to select the appropriate set of mRNAs and miRNAs for cancer tissue classification, whereas Mukhopadhyay et al. [20] used a multiobjective evolutionary algorithm (MOGA) technique with SVM as a wrapper to select appropriate miRNAs for classification of normal and tumor tissues.

All these studies suffer from the same drawback, *i.e.*, use of only one single classifier like SVM [18], while no single classifier is suitable for solving different classification problems. Some classifiers perform well for some domains whereas others perform well for some other domains. Therefore, it is pivotal to automatically select the classifier from a set of classifiers for a particular classification problem. Moreover, automatic selection of feature and parameter combination corresponding to the selected classifier is also necessary. However, none of the existing approaches [16], [19], [20] provides a way to combine the set of solutions obtained after the application of any multi-objective optimization-based technique.

In the current study, we considered the problem of automatic classifier selection and the corresponding feature and parameter combination selection as a multi-objective optimization problem [17]. We thus developed a multi-objective optimization-based two-stage algorithm and evaluated its performance using real SPECT dataset and miRNA and mRNA expression datasets.

## Method

### Formulation of the proposed approach using multiobjective optimization

An automatic approach was proposed for selecting a set of classifiers from a group of classifiers, by optimizing precision, recall, and number of selected features simultaneously using a multiobjective optimization technique. The selected classifiers are then applied to the datasets to obtain the final result using stack-based ensemble technique. The proposed methodology comprises two stage approaches, which are explained below.

#### First stage

We used the search capability of a popular multi-objective optimization technique, non-dominated sorting genetic algorithm-II (NSGA-II) [17] to determine the appropriate classifier type, parameter combination and feature combination from a given classification problem. The basic steps of NSGA-II are shown in Figure S1.

##### String representation

**Figure 1:**
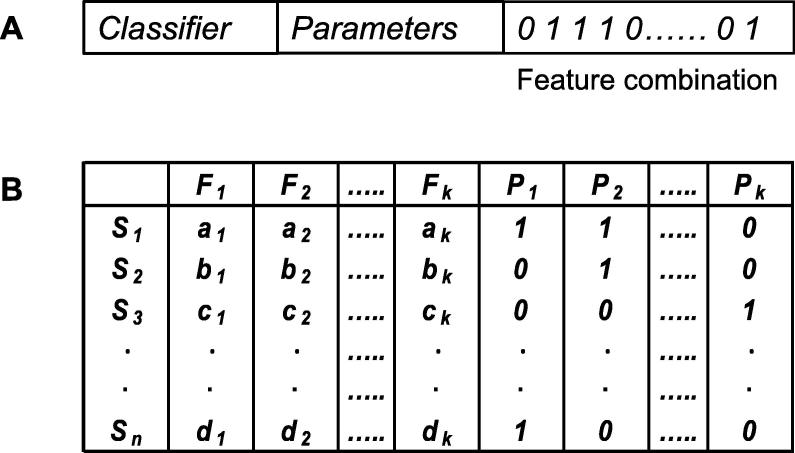
**Two stages of the proposed NSGA-II-based approach** **A.** String/solution representation showing the first stage. There are three parts involved, including the type of classifier, parameters corresponding to the selected classifier, and feature combination. **B.** Stack-based ensemble showing the second stage of the proposed approach. *S*_1_, *S*_2_, …, *S_n_* represent the samples present in the dataset; *F*_1_, *F*_2,_ …, *F_k_* represent the corresponding features; *P*_1_, *P*_2,_ …, *P_k_* represent the predicted class labels corresponding to a particular classifier. The absence and presence of a particular feature are indicated with “0” and “1”, respectively. NSGA-II, non-dominated sorting genetic algorithm-II.

Individuals or strings are used as inputs to NSGA-II-based approach. A string encodes a possible solution for the given problem. As there are three subcomponents of the given problem, the string is represented in three parts, *i.e.*, classifier type, parameter, and feature combinations (Figure 1).

The first part represents the type of the classifier used. In the current study, four classifiers were used, including random forest (RF), random tree (RT), sequential minimal optimization (SMO), and logistic regression (LR) [21]. Any one of these four classifiers is present in a particular solution, with values 1, 2, 3, and 4 representing RF, RT, SMO, and LR, respectively.

The second part of the string/solution contains the parameters corresponding to a specific classifier. Parameters used in RF include the number of trees (possible values: 10, 20, and 30) and the number of features (possible values: 0, 5, and 6). Parameters used in RT include the minimum total weight of instance in a leaf (possible values: 1.0, 1.05, and 1.25) and the number of randomly-chosen features (possible values: 0, 3, and 7). Only a single parameter of SMO, complexity, was considered in our algorithm (possible values: 1, 3, and 8). No particular parameter values were determined for LR; instead, the default values are used.

Finally the third component of the solution/string represents features in the form of binary string where “0” and “1” indicate the absence and presence of a particular feature, respectively.

##### Population initialization

Initialization of all strings is automatically performed. The first part can randomly select values 1, 2, 3, or 4 to represent RF, RT, SMO, and LR, respectively. The second part of the string is initialized by selecting random values from the given set to assign the parameter values corresponding to a particular classifier. For example, if the first part of the string contains the value 3 (SMO), then a parameter value of 1, 3, or 8 can be randomly selected from the set. Finally, the third part is initialized with values “0” or “1”. If the dataset contains total *N* features, then each feature position is initialized to 0 or 1. Thus a binary string of size *N* is generated.

##### Objective function calculation

Let (*S*) denote a set of features whose values are “1” in the feature part of the string/solution (third part). The encoded classifier and its parameter and feature combinations (*S*) are obtained. The selected classifier, along with selected parameter and feature combinations, is executed on the available dataset using leave-one-out cross-validation (LOOCV). Two classification quality measures, average precision [22] and recall [22] values are calculated and used as the first two objective functions whose values are to be maximized (higher values of precision and recall correspond to good classification qualities). The third one, *i.e.*, the number of features (S), is to be minimized. The objective of the current work is to select that particular classifier which provides good performance (with respect to recall and precision) with minimum number of features.

##### Genetic operators

Three mutation operators are defined to obtain more diversified solutions. Type 1 is for changes present in the whole string, type 2 is for changes present in the parameter and feature combinations, while type 3 is for changes present only in the feature combination. Any of the above mentioned mutation operations are applied to a particular string at a given generation. The other operations of NSGA-II are applied to explore the search space judiciously. Another search operation, namely crossover operation that is used to exchange information between two given solutions is applied only on the feature part of the string using normal single point crossover operator.

##### Termination criterion

The process of fitness computation, selection, crossover, and mutation is executed for the fixed number of generations (100 iterations by default). At the last iteration of NSGA-II, a set of non-dominated solutions (containing type of classifier with its selected parameters and features that is to be applied on the datasets) is provided in the ranking order, with “rank 1” being the highest.

#### Second stage

Outputs of all the “rank 1” solutions obtained from the first stage were combined in the second stage. Unique solutions from the solution set were retrieved; each unique solution represents a particular classifier type with a set of features and parameter combinations. This classifier is executed on the training dataset to predict the class label of each data sample. The class labels are converted to “0” and “1” for binary classification problem, with “1” representing the positive class (normal type) and “0” representing the negative class (tumor type).

For multiple-class problem one-vs.-rest strategy is used. This strategy involves training a single classifier per class, with the samples of that class as positive samples, “1” and all other samples as negatives, “0”.

**Figure 2:**
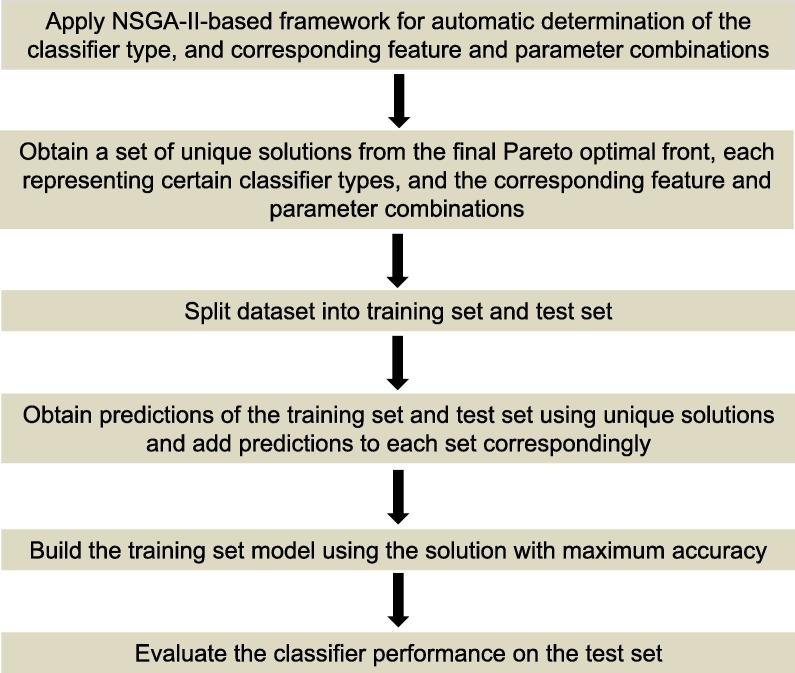
**Steps of the proposed method**

If *N* unique solutions are present in solution set from stage 1, totally *N* predictions would be available for each sample of the training set. These *N* predictions are added as the feature values of that particular sample of the training set. In this way, we generate a new training dataset. Similarly a new test dataset is also generated by adding the predictions of *N* classifiers to the available feature combinations for all the samples. Now a new classifier (selected classifier with the highest *F*-measure value from the first-stage) is executed on the newlygenerated training set to build the model, and the generated model is tested on the new test set. The accuracy of the final test set is calculated and used as the final accuracy of the combined solutions. The stack-based ensemble approach and the complete procedure of our algorithm are shown in Figure 1 and Figure 2, respectively.

### Datasets

We used five datasets, GCM miRNA, GCM mRNA, GCM miRNA_217, SRBCT, and brain tumor (POM) to evaluate the performance of the proposed method. The first three datasets are mammalian cancer datasets downloaded from http://portals.broadinstitute.org/cgi-bin/cancer/datasets.cgi. Total 11 datasets containing 89 samples of both normal and tumor types were extracted from GCM mRNA dataset [23], with each sample having 16,063 mRNAs (*i.e.*, features). Similarly, 11 datasets containing 89 samples of both normal and tumor types were extracted from GCM miRNA dataset [23], with each sample having 217 miRNAs (*i.e.*, features). The original dataset for GCM miRNA_217 expression profiles’ dataset [23] contains 218 samples of both normal and tissue types. Each sample contains 217 miRNAs from different tumor types. From this dataset, 6 datasets were extracted containing samples from kidney, colon, prostate, lung, breast, and uterus; thus giving a total of 75 samples of both normal and tissue types. The datasets are divided into 60% samples in the training set and 40% samples in the test set, and the class distributions are kept uniform in both sets.

The SPECT dataset was obtained from https://archive.ics.uci.edu/ml/datasets/spect+heart and the complete dataset is divided into two sets. The training set and test set contained 80 and 187 samples of both benign and malign classes, respectively, with each sample having 44 features. The brain tumor dataset (POM dataset) was obtained from http://www.gems-system.org/, which contains 90 samples with each sample having 5921 features. The training set and test set of POM dataset contained 42 and 48 samples, respectively.

### Data preprocessing

SPECT dataset was directly used for analysis due to the limited number of features. Data preprocessing was performed to reduce the dimensionality of feature space for the GCM miRNA, GCM mRNA, and POM datasets, since the number of available features is 217, 16,063, and 5921, respectively. We used “Chi squared attribute evaluation” as a feature selection algorithm [24], which evaluates the features by calculating the *Χ*^2^ values of all the features. The *Χ*^2^ values are sorted in the descending order and features with top 100 highest *Χ*^2^ values are used in our algorithm.

For GCM miRNA 217 dataset, different data preprocessing steps are applied. Each sample is first normalized to have variance = 1 and mean = 0. The signal-to-noise-ratio (SNR) was used as the initial filtering method to reduce the miRNA feature set. SNR is given by

$$\text{SNR}=\frac{\mu_{1}-\mu_{2}}{\sigma_{2}-\sigma_{2}}$$

where *µ*_1_ and *µ*_2_ denote the means, and *σ*_1_ and *σ*_2_ denote the standard deviations of normal and tumor classes for the corresponding miRNA, respectively. SNR values of 217 miRNAs were ranked in a decreasing order and miRNAs with SNR value ≥the mean of all absolute SNR values were retained. As a result, 99 out of 217 miRNAs (*i.e.*, features) are used in our proposed algorithm.

## Results and discussion

We applied our proposed multiobjective optimization based algorithm on the given datasets for fixed number of generations. The parameters for our proposed algorithm are as follows: initial population size = 52, number of generations = 50, mutation probability = 0.1, and crossover probability = 0.9. Three quality measures, namely precision, recall, and number of features, are used for performance evaluation. Good classification corresponds to high recall and precision values and low number of features. Weka was adopted for classification [21], since it is an easy and simple tool to use and contains all the classifiers used in our proposed algorithm. Default parameters are used for SVM-nRFE as described previously [19].

### Performance analysis

The final obtained accuracies for all the datasets examined are shown in Table 1. Accuracy for SPECT dataset was 95.87%, whereas accuracies for the three GCM datasets were more than 97%. The high accuracies for the latter datasets could be due to fewer samples included in these datasets than those in the SPECT dataset. However, compared to the SPECT dataset, lower accuracy was found for POM dataset in spite of having fewer instances, because there are more classes in POM dataset (5 classes) than in SPECT dataset (2 classes).

**Table 1:** **Performance comparison between the proposed approach and SVM-nRFE**

**Dataset**	**No. of samples**	**No. of features**	**Our proposed approach**					**Accuracy (%) of SVM-nRFE**
			**Precision**	**Recall**	**No. of features used**	**No. of unique solutions obtained**	**Accuracy (%)**	
SPECT	267	44	0.9489	1.0000	17	18	95.87	87.30
GCM miRNA	89	100	0.9350	0.9597	13	6	97.43	95.80
GCM mRNA	89	100	0.9670	0.9804	16	7	97.14	94.60
GCM miRNA 217	75	99	0.9460	0.9780	12	8	97.11	88.30
POM	90	100	0.8630	0.8400	23	20	84.00	76.00

*Note*: SVM-nRFE, support vector machine-based recursive feature elimination technique.

Table 1 also shows the number of unique solutions obtained from the best population (good convergence and less diversity) in the first stage of the proposed approach for each dataset. The diversified unique solutions (good convergence and good diversity) obtained in the first stage for GCM miRNA 217 and GCM mRNA datasets are shown in Table S1 and Table S2, respectively. As shown in Table S1, there are 8 unique solutions with different classifiers and parameter combinations for the GCM miRNA 217. These solutions are obtained from the set of solutions whose ranks were “1” in the final population obtained from the first stage.

### Comparative study

To further demonstrate the effectiveness of our proposed algorithm, we compared our algorithm with several algorithms like MOGA [20], RPSGAe [16], SVM-nRFE [19], SCAD [25], LASSO [26], and ranksum [27], [28] for various available datasets.

As shown in Table 1, higher accuracies were achieved using our algorithm than using SVM-nRFE approach [19] for all the datasets tested. For SPECT dataset, we also compared the performance of our algorithm with RPSGAe approach using *F*-measure. As a result, *F*-measure was 96.80% using our method as compared to 95.10% using RPSGAe [16], indicating that our algorithm in general performs better than RPSGAe [16]. Furthermore, we compared our algorithm with several available algorithms for GCM miRNA 217 dataset and found that our proposed approach performed better than all the existing methods examined in terms of accuracy (Table S3).

A set of solutions (Pareto-optimal set) containing different classifier types and different parameter and feature combinations were generated in the first stage of our algorithm. The minimum and maximum numbers of features selected by the solutions on the final Pareto optimal front are shown in Table S4.

The number of features and the accuracy were used as the criteria to select unique solutions. Given the trade-off between the number of features and accuracy, we considered the feature set with maximum accuracy but the minimum possible number of features. We found that accuracies obtained for the three GCM datasets were much higher than that for SPECT dataset (>90% for GCM datasets vs. 75.40% for SPECT) (Table S5).

The accuracy could be further improved by combining solutions obtained from both the first stage and the second stage. For all the cases, performance was further improved in the second stage over the first stage (comparing the accuracies in Table S5 and Table 1), demonstrating the benefit of combining the solutions obtained from the first stage using a stacked-based ensemble approach.

### Biological significance

Features reported in Table S5 are used to analyze the biological significance of the selected miRNAs corresponding to GCM miRNA and GCM miRNA 217 datasets. We first determined the number of mRNAs targeted by the miRNAs obtained from the first stage of our algorithm for these datasets, using miRDB database (version 5.0; http://mirdb.org/) for target prediction. Human cancer miRNA network [29] was also employed to find cancer types associated with the corresponding miRNAs. Table 2 and Table 3 report the number of mRNA targets and cancer types associated with each selected miRNA corresponding to GCM miRNA and GCM miRNA 217 datasets, respectively. GCM_miRNA dataset is a mammalian dataset, hence mouse miRNAs initially got selected by the algorithm were not further analyzed. As a result, only 9 out of 11 selected miRNAs are considered for GCM miRNA dataset (Table 2). Similarly, the three mouse miRNAs were not further analyzed for the miRNA 217 dataset. In addition, no cancer type was listed for miRNA hsa-miR-220 (Table 3), which is no longer considered as a miRNA [30].

**Table 2:** **Number of mRNA targets and cancer types associated with the selected miRNAs for the GCM miRNA dataset**

**No.**	**miRNA**	**No. of mRNA targets**	**Cancer type associated**
1	hsa-miR-18	682	HCC/liver, lung, follicular lymphoma
2	hsa-miR-101	671	Breast, lung, ovary
3	hsa-miR-126^*^	644	Colon, CNS, lung, hematologic, HCC/liver
4	hsa-miR-30d	1603	CNS
5	hsa-miR-30a	1609	Lung
6	hsa-miR-152	559	Colon, hematologic
7	hsa-miR-148	945	Pancreas
8	hsa-miR-185	1517	Bladder, kidney
9	hsa-miR-199a^*^	621	Colon, HCC/liver, hematologic
10	mmu-miR-342	542	–
11	mmu-miR-340	538	–

*Note*: Data were generated based on the data obtained in [29], which is a mammalian dataset. HCC, hepatocellular carcinoma; CNS, central nervous system.

**Table 3:** **Number of mRNA targets and cancer types associated with the selected miRNAs for the GCM miRNA 217 dataset**

**No.**	**miRNA**	**No. of mRNA targets**	**Cancer type associated**
1	hsa-miR-99a	41	Colon, lung, uterus, hematologic
2	hsa-miR-197	436	CNS, thyroid, uterus
3	hsa-miR-220	–	–
4	hsa-miR-195	1497	CLL, CNS, HCC/liver, lung, hematologic, uterus
5	hsa-miR-154	373	CNS
6	hsa-miR-184	45	Uterus
7	hsa-miR-133a	310	Bladder, breast
8	hsa-miR-32	880	Colon, pancreas, lung, prostate, uterus
9	mmu-miR-292	497	–
10	mmu-miR-293^*^	266	–
11	mmu-miR-339	256	–

*Note*: GCM miRNA217 dataset was generated based on the data obtained in [29], which is a mammalian dataset. HCC, hepatocellular carcinoma; CNS, central nervous system; CLL, chronic lymphocytic leukemia.

To identify the biological activities associated with the miRNAs selected using our approach, we performed KEGG pathway enrichment analysis of the target genes using the database for annotation, visualization and integrated discovery (DAVID; http://david.abcc.ncifcrf.gov). The KEGG pathways of the obtained miRNAs with cancer types associated (Table 2 and Table 3), along with their *P* values for GCM miRNA dataset and GCM miRNA 217 dataset, are shown in Table 4 and Table 5, respectively. It was found that the term “pathways in cancer” was present most frequently in the selected pathways. Moreover, specific cancer pathways also appeared as the significant pathways for the individual miRNA markers. For example, hsa-miR-126^∗^ is involved in colorectal cancer pathway; hsa-miR-30d and hsa-miR-30a have target genes involved in the pathway of renal cell carcinoma and prostate cancer; similarly, hsa-miR-32 is involved in small-cell lung cancer pathway. These data indicate that the selected miRNAs are associated with different cancer pathways and thus can be potentially considered as miRNA markers for cancer.

**Table 4:** **Top significant KEGG pathways identified for the GCM miRNA dataset**

**No.**	**miRNA**	**KEGG pathway**	***P* value**
1	hsa-miR-18	Gap junction Apoptosis Endocytosis Hedgehog signaling pathway Vascular smooth muscle contraction	5.2E−3 1.8E−2 3.2E−2 4.4E−2 5.1E−2

2	hsa-miR-101	Ubiquitin mediated proteolysis Lysosome	8.8E−3 5.4E−2

3	hsa-miR-126^*^	Melanogenesis Wnt signaling pathway Long-term potentiation Pathways in cancer Colorectal cancer	1.5E−2 1.9E−2 2.0E−2 2.2E−2 2.9E−2

4	hsa-miR-30d	Ubiquitin mediated proteolysis Renal cell carcinoma Prostate cancer Long-term potentiation Pathways in cancer	2.1E−3 3.0E−3 4.8E−3 6.3E−3 2.4E−2

5	hsa-miR-30a	Ubiquitin mediated proteolysis Renal cell carcinoma Prostate cancer Long-term potentiation Pathways in cancer	1.8E−3 2.8E−3 4.4E−3 5.8E−2 2.1E−2

6	hsa-miR-152	Ubiquitin mediated proteolysis Neurotrophin signaling pathway MAPK signaling pathway Axon guidance Pathways in cancer	7.7E−4 2.5E−3 6.0E−3 8.7E−3 2.0E−2

7	hsa-miR-148	Ubiquitin mediated proteolysis Wnt signaling pathway B cell receptor signaling pathway Prostate cancer Pathways in cancer	2.8E−4 1.1E−2 1.2E−2 2.1E−2 4.1E−2

8	hsa-miR-185	Axon guidance Ubiquitin mediated proteolysis ErbB signaling pathway Wnt signaling pathway Long-term depression	4.5E−5 1.3E−4 3.5E−3 6.5E−3 6.6E−3

9	hsa-miR-199a^*^	Axon guidance Renal cell carcinoma Ubiquitin mediated proteolysis Pathways in cancer ErbB signaling pathway	1.3E−4 3.2E−4 3.5E−4 9.9E−4 3.7E−3

**Table 5:** **Top significant KEGG pathways identified for the GCM miRNA_217 dataset**

**No.**	**miRNA**	**KEGG pathway**	***P* value**
1	hsa-miR-99a	MAPK signaling pathway Type II diabetes mellitus	7.7E−2 8.0E−2

2	hsa-miR-197	MAPK signaling pathway Vascular smooth muscle contraction Pathways in cancer Adherens junction Regulation of actin cytoskeleton	4.4E−3 5.1E−3 1.8E−2 2.4E−2 3.4E−2

3	hsa-miR-195	Pathways in cancer Focal adhesion Neurotrophin signaling pathway p53 signaling pathway Cell cycle	1.1E−3 5.5E−3 1.3E−2 1.4E−2 1.4E−2

4	hsa-miR-154	Ubiquitin-mediated proteolysis Wnt signaling pathway TGF-beta signaling pathway	4.8E−3 5.8E−2 6.4E−2

5	has-miR-184	Neurodegenerative diseases Long-term potentiation Phosphatidylinositol signaling system Melanogenesis	1.3E−3 3.0E−3 1.6E−2 5.1E−4

6	hsa-miR-133a	Seleno amino acid metabolism Long-term depression Cysteine and methionine metabolism	3.3E−2 4.0E−2 5.3E−2

7	hsa-miR-32	RNA degradation Biosynthesis of unsaturated fatty acids Small cell lung cancer Pantothenate and Cao biosynthesis Wnt signaling pathway	1.1E−2 2.9E−2 3.7E−2 4.4E−2 6.5E−2

## Conclusions

In this article, we developed a two-stage approach to solve some real-life classification problems. Four algorithms are used for classification and three performance criteria are optimized simultaneously to select the better solutions obtained from the set of solutions. We tested our algorithm on five real-life datasets to evaluate its performance. The obtained results show the superior effectiveness of the proposed approach to several existing methods examined.

## Authors’ contributions

SS conceived the idea and developed the algorithm. RY implemented the approach, pre-processed the datasets, and evaluated the approach on some datasets. SM analyzed the results and performed statistical and biological significance tests. All authors were involved in manuscript writing, read and approved the final manuscript.

## Competing interests

The authors have declared no competing interests.

## Supplementary material

Supplementary Figure S1 **NSGA-II procedure** Step 1: tournament. Each intermediate solution is compared with another randomly-selected intermediate solution (niche comparison). Copy of winner is placed in mating pool. Step 2: crossover. Crossover operation is applied on two parents. Offsprings generated from crossover are placed in offspring population Qt + 1. Step 3: non-dominated sorting. Non-dominated sorting is applied to Rt. All non-dominated fronts of Pt + Qt are copied to parent population rank by rank denoted by Rt. Step 4: crowded distance sorting. Addition of individuals is stopped when size of parent population is larger than population size *N*. Individuals in the last rank that make parent population greater than *N* are sorted by crowded distance technique. NSGA-II, non-dominated sorting genetic algorithm-II; P*t*, parent population at generation *t*; P*t* + 1, parent population that generates offspring to the next generation; Q*t*, offspring generated from P*t*.

Supplementary Table S1 **Diversified unique solutions obtained for the GCM miRNA 217 dataset in the first stage of the proposed approach**

Supplementary Table S2 **Diversified unique solutions obtained for the GCM mRNA dataset in the first stage of the proposed approach**

Supplementary Table S3 **Performance comparison between the proposed approach and other feature selection methods for the GCM miRNA 217 dataset**

Supplementary Table S4 **Number of features present in the unique solutions obtained for the five datasets using the proposed approach**

Supplementary Table S5 **Accuracies and classifiers selected corresponding to the minimum feature combination determined in the first stage of the proposed approach for the five datasets**

## References

[1] Stewart B.W., Kleihues P. (2003). World cancer report.

[2] Lv M., Zhu X., Chen W., Zhao J., Tang J. (2013). Searching for candidate microRNA biomarkers in detection of breast cancer: a meta-analysis. Cancer Biomark.

[3] Mishra P.J. (2014). MicroRNAs as promising biomarkers in cancer diagnostics. Biomark Res.

[4] Ren A., Dong Y., Tsoi H., Yu J. (2015). Detection of miRNA as non-invasive biomarkers of colorectal cancer. Int J Mol Sci.

[5] Wu X., Somlo G., Yu Y., Palomares M.R., Li A.X., Zhou W. (2012). *De novo* sequencing of circulating miRNAs identifies novel markers predicting clinical outcome of locally advanced breast cancer. J Transl Med.

[6] Gambari R., Fabbri E., Borgatti M., Lampronti I., Finotti A., Brognara E. (2011). Targeting microRNAs involved in human diseases: a novel approach for modification of gene expression and drug development. Biochem Pharmacol.

[7] Fu S.W., Chen L., Man Y. (2011). miRNA biomarkers in breast cancer detection and management. J Cancer.

[8] Etheridge A., Lee I., Hood L., Galas D., Wang K. (2011). Extracellular microRNA: a new source of biomarkers. Mutat Res.

[9] Jacobsen A., Silber J., Harinath G., Huse J.T., Schultz N., Sander C. (2013). Analysis of microRNA-target interactions across diverse cancer types. Nat Struct Mol Biol.

[10] Wei M.M., Zhou G.B. (2016). Long non-coding RNAs and their roles in non-small-cell lung cancer. Genomics Proteomics Bioinformatics.

[11] Yang Y., Dong X., Xie B., Ding N., Chen J., Li Y. (2015). Databases and web tools for cancer genomics study. Genomics Proteomics Bioinformatics.

[12] Chakraborty C., Chin K.Y., Das S. (2016). miRNA-regulated cancer stem cells: understanding the property and the role of miRNA in carcinogenesis. Tumour Biol.

[13] Yang Q., Diamond M.P., Al-Hendy A. (2016). The emerging role of extracellular vesicle-derived miRNAs: implication in cancer progression and stem cell related diseases. J Clin Epigenet.

[14] Liu H., Yu L. (2005). Toward integrating feature selection algorithms for classification and clustering. IEEE Trans Knowl Data Eng.

[15] Blum A.L., Langley P. (1997). Selection of relevant features and examples in machine learning. Artif Intell.

[16] Gaspar-Cunha A. (2010). Feature selection using multi-objective evolutionary algorithms: application to cardiac SPECT diagnosis. Adv Bioinformatics.

[17] Deb K., Pratap A., Agarwal S., Meyarivan T. (2002). A fast and elitist multiobjective genetic algorithm: NSGA-II. IEEE Trans Evol Comput.

[18] Zhang X. (2000). Introduction to statistical learning theory and support vector machines. Acta Automatica Sinica.

[19] Peng S., Zeng X., Li X., Peng X., Chen L. (2009). Multi-class cancer classification through gene expression profiles: microRNA versus mRNA. J Genet Genomics.

[20] Mukhopadhyay A., Maulik U. (2013). An SVM-wrapped multiobjective evolutionary feature selection approach for identifying cancer-microRNA markers. IEEE Trans Nanobioscience.

[21] Bishop C.M. (2006). Pattern recognition and machine learning.

[22] Olson D.L., Delen D. (2008). Advanced data mining techniques.

[23] Lu J., Getz G., Miska E.A., Alvarez-Saavedra E., Lamb J., Peck D. (2005). MicroRNA expression profiles classify human cancers. Nature.

[24] Forbes C., Evans M., Hastings N., Peacock B. (2011). Statistical distributions.

[25] Fan J., Li R. (2001). Variable selection via nonconcave penalized likelihood and its oracle properties. J Am Stat Assoc.

[26] Tibshirani R. (1996). Regression shrinkage and selection via the lasso. J R Stat Soc Series B Stat Methodol.

[27] Bickel P.J., Doksum K.A. (2015). Mathematical statistics: basic ideas and selected topics.

[28] Schucany W.R., Randles R.H., Wolfe D.A. (1981). Introduction to the theory of nonparametric statistics. SIAM Rev Soc Ind Appl Math.

[29] Bandyopadhyay S., Mitra R., Maulik U., Zhang M.Q. (2010). Development of the human cancer microRNA network. Silence.

[30] Chiang H.R., Schoenfeld L.W., Ruby J.G., Auyeung V.C., Spies N., Baek D. (2010). Mammalian microRNAs: experimental evaluation of novel and previously annotated genes. Genes Dev.

