# Supplementary material for: A Stack-based Ensemble Framework for Detecting Cancer MicroRNA Biomarkers
Source: Genomics Proteomics Bioinformatics. 2017 Dec 12;15(6):381–8. doi: 10.1016/j.gpb.2016.10.006 (PMC5828659; doi:10.1016/j.gpb.2016.10.006)
Supplement: Supplementary Figure S1 — NSGA-II procedure Step 1: tournament. Each intermediate solution is compared with another randomly-selected intermediate solution (niche comparison). Copy of winner is placed in mating pool. Step 2: crossover. Crossover operation is applied on two parents. Offsprings generated from crossover are placed in offspring population Qt + 1. Step 3: non-dominated sorting. Non-dominated sorting is applied to Rt. All non-dominated fronts of Pt + Qt are copied to parent population rank by rank denoted by Rt. Step 4: crowded distance sorting. Addition of individuals is stopped when size of parent population is larger than population size N. Individuals in the last rank that make parent population greater than N are sorted by crowded distance technique. NSGA-II, non-dominated sorting genetic algorithm-II; Pt, parent population at generation t; Pt + 1, parent population that generates offspring to the next generation; Qt, offspring generated from Pt. [file mmc1.pptx]

## Slide 1
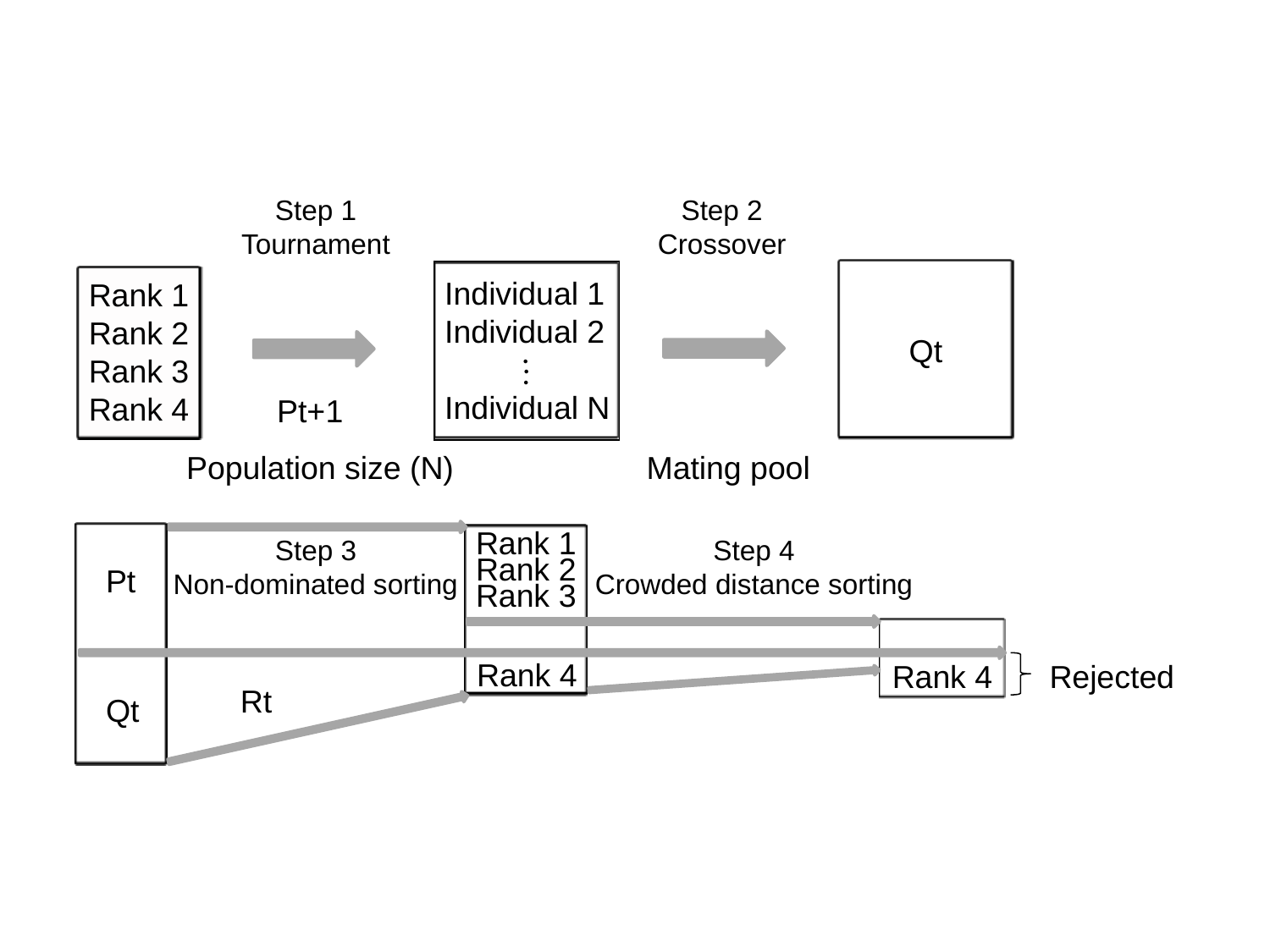

Step 1
Tournament
Step 2
Crossover
Rank 1
Rank 2
Rank 3
Rank 4
Individual 1
Individual 2
Individual N
Qt
.
.
.
Pt+1
Population size (N)
Mating pool
Step 3
Non-dominated sorting
Step 4
Crowded distance sorting
Rank 1
Rank 2
Rank 3
Pt
Rank 4
Rejected
Rank 4
Rt
Qt
