# Supplementary material for: A Stack-based Ensemble Framework for Detecting Cancer MicroRNA Biomarkers
Source: Genomics Proteomics Bioinformatics. 2017 Dec 12;15(6):381–8. doi: 10.1016/j.gpb.2016.10.006 (PMC5828659; doi:10.1016/j.gpb.2016.10.006)
Supplement: Supplementary Table S1 — Diversified unique solutions obtained for the GCM miRNA 217 dataset in the first stage of the proposed approach [file mmc2.docx]

**Table S1 Diversified unique solutions obtained for the GCM miRNA 217 dataset in the first stage of the proposed approach**

| **Classifier** | **Parameter 1** | **Parameter 2** | **No. of features** |
| --- | --- | --- | --- |
| Sequential minimal optimization | 3.0 | NA | 11 |
| Random forest | 30 | 5 | 11 |
| Random tree | 7 | 1.05 | 13 |
| Random forest | 20 | 5 | 13 |
| Random forest | 30 | 5 | 13 |
| Random forest | 20 | 6 | 11 |
| Random tree | 7 | 1.00 | 13 |
| Random forest | 20 | 6 | 13 |

*Note*: Parameter 1 refers to the number of trees (random forest), minimum total weight of instance in a leaf (random tree), or complexity (sequential minimal optimization), whereas parameter 2 refers to the number of features (random forest) or number of randomly chosen features (random tree). Sequential minimal optimization and random forest performed better than logistic regression on the GCM　miRNA 217 dataset; therefore data for LR were not reported. NA, not applicable.
