# Supplementary material for: A Stack-based Ensemble Framework for Detecting Cancer MicroRNA Biomarkers
Source: Genomics Proteomics Bioinformatics. 2017 Dec 12;15(6):381–8. doi: 10.1016/j.gpb.2016.10.006 (PMC5828659; doi:10.1016/j.gpb.2016.10.006)
Supplement: Supplementary Table S2 — Diversified unique solutions obtained for the GCM mRNA dataset in the first stage of the proposed approach [file mmc3.docx]

**Table S2 Diversified unique solutions obtained for the GCM mRNA dataset in the first stage of the proposed approach**

| **Classifier** | **Parameter 1** | **Parameter 2** | **No. of features** |
| --- | --- | --- | --- |
| Sequential minimal optimization | 3.0 | NA | 12 |
| Sequential minimal optimization | 8.0 | NA | 26 |
| Random forest | 20 | 5 | 14 |
| Sequential minimal optimization | 8.0 | NA | 18 |
| Sequential minimal optimization | 3.0 | NA | 35 |
| Logistic regression | NA | NA | 12 |
| Sequential minimal optimization | 8.0 | NA | 12 |

*Note*: Parameter 1 refers to the number of trees (random forest) or complexity (sequential minimal optimization), whereas parameter 2 refers to the number of features for random forest. Default parameters are used for logistic regression. NA, not applicable.
