# Supplementary material for: A Stack-based Ensemble Framework for Detecting Cancer MicroRNA Biomarkers
Source: Genomics Proteomics Bioinformatics. 2017 Dec 12;15(6):381–8. doi: 10.1016/j.gpb.2016.10.006 (PMC5828659; doi:10.1016/j.gpb.2016.10.006)
Supplement: Supplementary Table S3 — Performance comparison between the proposed approach and other feature selection methods for the GCM miRNA 217 dataset [file mmc4.docx]

**Table S3 Performance comparison between the proposed approach and other feature selection methods for the GCM miRNA 217 dataset**

| **Method** | **Accuracy (%)** | **Ref.** |
| --- | --- | --- |
| Our proposed approach | 97.14 | – |
| MOGA | 89.80 | [20] |
| SNR | 87.76 | [24] |
| SCAD | 85.71 | [25] |
| LASSO | 87.76 | [26] |
| *t*-test | 85.71 | [27] |
| ranksum | 87.76 | [28] |

*Note*: MOGA, multi-objective genetic algorithm; SNR, signal-to-noise ratio; SCAD, simultaneous clustering and attribute discrimination.
