# Supplementary material for: A Stack-based Ensemble Framework for Detecting Cancer MicroRNA Biomarkers
Source: Genomics Proteomics Bioinformatics. 2017 Dec 12;15(6):381–8. doi: 10.1016/j.gpb.2016.10.006 (PMC5828659; doi:10.1016/j.gpb.2016.10.006)
Supplement: Supplementary Table S4 — Number of features present in the unique solutions obtained for the five datasets using the proposed approach [file mmc5.docx]

**Table S4 Number of features present in the unique solutions obtained for the five datasets using the proposed approach**

| **Dataset** | **Minimum No. of features** | **Maximum No. of features** |
| --- | --- | --- |
| SPECT | 4 | 18 |
| GCM miRNA | 9 | 16 |
| GCM mRNA | 12 | 26 |
| GCM miRNA 217 | 11 | 13 |
| POM | 17 | 27 |
