# Supplementary material for: A Stack-based Ensemble Framework for Detecting Cancer MicroRNA Biomarkers
Source: Genomics Proteomics Bioinformatics. 2017 Dec 12;15(6):381–8. doi: 10.1016/j.gpb.2016.10.006 (PMC5828659; doi:10.1016/j.gpb.2016.10.006)
Supplement: Supplementary Table S5 — Accuracies and classifiers selected corresponding to the minimum feature combination determined in the first stage of the proposed approach for the five datasets [file mmc6.docx]

| **Dataset** | **No. of features** | **Accuracy (%)** | **Classifier** | **Parameters** |
| --- | --- | --- | --- | --- |
| SPECT | 6 | 75.40 | Logistic regression | – |
| GCM miRNA | 11 | 92.30 | Random tree | 0, 1 |
| GCM mRNA | 14 | 94.87 | Random forest | 20, 5 |
| GCM miRNA 217 | 11 | 94.28 | Random forest | 30, 5 |
| POM | 17 | 77.85 | Sequential minimal optimization | 3 |

**Table S5 Accuracies and classifiers selected corresponding to the minimum feature combination determined in the first stage of the proposed approach for the five datasets**
